# Osteoporosis: interferon-gamma-mediated bone remodeling in osteoimmunology

**DOI:** 10.3389/fimmu.2024.1396122

**Published:** 2024-05-16

**Authors:** Siying Li, Gang Liu, Siwang Hu

**Affiliations:** ^1^ The Orthopaedic Center, The First People’s Hospital of Wenling, Taizhou University Affiliated Wenling Hospital, Wenling, Zhejiang, China; ^2^ College of Bioscience and Biotechnology, Hunan Agricultural University, Changsha, Hunan, China

**Keywords:** osteoporosis, bone remodeling, interferon-gamma, osteoblast, osteoclast

## Abstract

As the world population ages, osteoporosis, the most common disease of bone metabolism, affects more than 200 million people worldwide. The etiology is an imbalance in bone remodeling process resulting in more significant bone resorption than bone remodeling. With the advent of the osteoimmunology field, the immune system’s role in skeletal pathologies is gradually being discovered. The cytokine interferon-gamma (IFN-γ), a member of the interferon family, is an important factor in the etiology and treatment of osteoporosis because it mediates bone remodeling. This review starts with bone remodeling process and includes the cellular and key signaling pathways of bone remodeling. The effects of IFN-γ on osteoblasts, osteoclasts, and bone mass are discussed separately, while the overall effects of IFN-γ on primary and secondary osteoporosis are summarized. The net effect of IFN-γ on bone appears to be highly dependent on the environment, dose, concentration, and stage of cellular differentiation. This review focuses on the mechanisms of bone remodeling and bone immunology, with a comprehensive discussion of the relationship between IFN-γ and osteoporosis. Finding the paradoxical balance of IFN-γ in bone immunology and exploring the potential of its clinical application provide new ideas for the clinical treatment of osteoporosis and drug development.

## Graphical Abstract

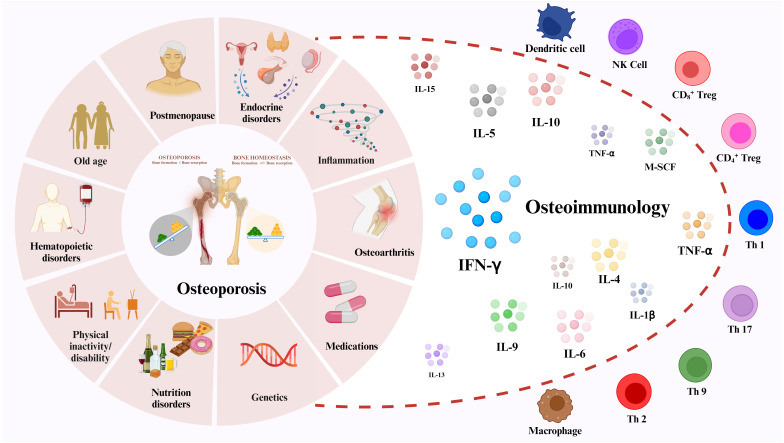

## 1 Introduction

Osteoporosis is one of the most common systemic bone diseases, characterized by bone mineral density (BMD) loss and bone tissue microstructural changes (1). This causes a reduction in bone mechanical strength, which increases the risk of fracture from the same impact or damage (2). Osteoporosis is usually categorized into two types: 1) primary osteoporosis often results from women’s postmenopause and aging, and 2) secondary osteoporosis can be caused by diseases or medications that affect bone metabolism, including diabetic osteoporosis (DOP) and glucocorticoid-induced osteoporosis (GIOP) (3, 4). According to the International Osteoporosis Foundation (IOF), osteoporosis is the fourth most common chronic disease after Inflammatory heart disease (IHD), Alzheimer’s disease (AD) and Lung cancer (5). According to statistics, osteoporosis affects 200 million people worldwide, mainly over 60. In addition, more than 8.9 million fractures are caused by osteoporosis each year, meaning that an osteoporotic fracture occurs every three seconds (5, 6). Patients with osteoporosis not only suffer from great pain and disability, but also impose a substantial financial burden on themselves and their families. As the world ages, osteoporosis becomes a public health problem.

Traditionally, osteoporosis is attributed to endocrine and metabolic disorders, along with external mechanical forces impacting bone remodeling processes (7, 8). In recent years, numerous studies have elucidated the intricate cross-talk between the immune system and the skeletal system. Given their shared microenvironment, the immune system exerts regulatory influence on bone cells via cytokines, chemokines, receptors, and transcription factors (9). The term “osteoimmunology” was first introduced by Takayanagi et al. in Nature in 2000 to delineate the intricate interplay between the immune and skeletal systems, emphasizing its significance (10). Over the past two decades, multiple studies have demonstrated the interaction between immune cells, cytokines, osteoblasts (OBs), and osteoclasts (OCs) in the regulation of bone remodeling, thereby influencing the pathogenesis of osteoporosis (11, 12).

Interferon plays a pivotal role as a key regulator of the immune response. Specifically, IFN-γ, an immune-derived cytokine, contributes to the regulation of the immune response in both innate and adaptive immune responses (13–15). Recent studies have shown that IFN-γ has a significant effect on the differentiation of OCs, OBs and bone marrow adipocytes (16–19). However, the specific role and mechanism of IFN-γ mediating osteoporosis through bone remodeling have not been fully explored. This review unfolds the mechanism of bone remodeling and related cells, highlighting the impact of IFN-γ on the development and function of OBs and OCs. It demonstrates IFN-γ-mediated primary and secondary osteoporosis, providing new ideas for OP treatment.

## 2 Osteoporosis and bone remodeling

The bone cells include OBs, OCs, osteocytes (OYs), hematopoietic stem cells (HSCs), mesenchymal stem cells (MSCs), stromal cells, etc. (20). Bone remodeling is a highly complex process that is influenced by a variety of factors and ultimately manifests itself in a fine-tuned balance between osteoblast-mediated bone formation and osteoclast-mediated bone resorption (21). However, the presence of various mediators and cytokines can disrupt this balance, leading to an increase in OC activity and subsequent reduction in BMD, ultimately culminating in the pathogenesis of osteoporosis.

### 2.1 Bone remodeling

**Figure 1:**
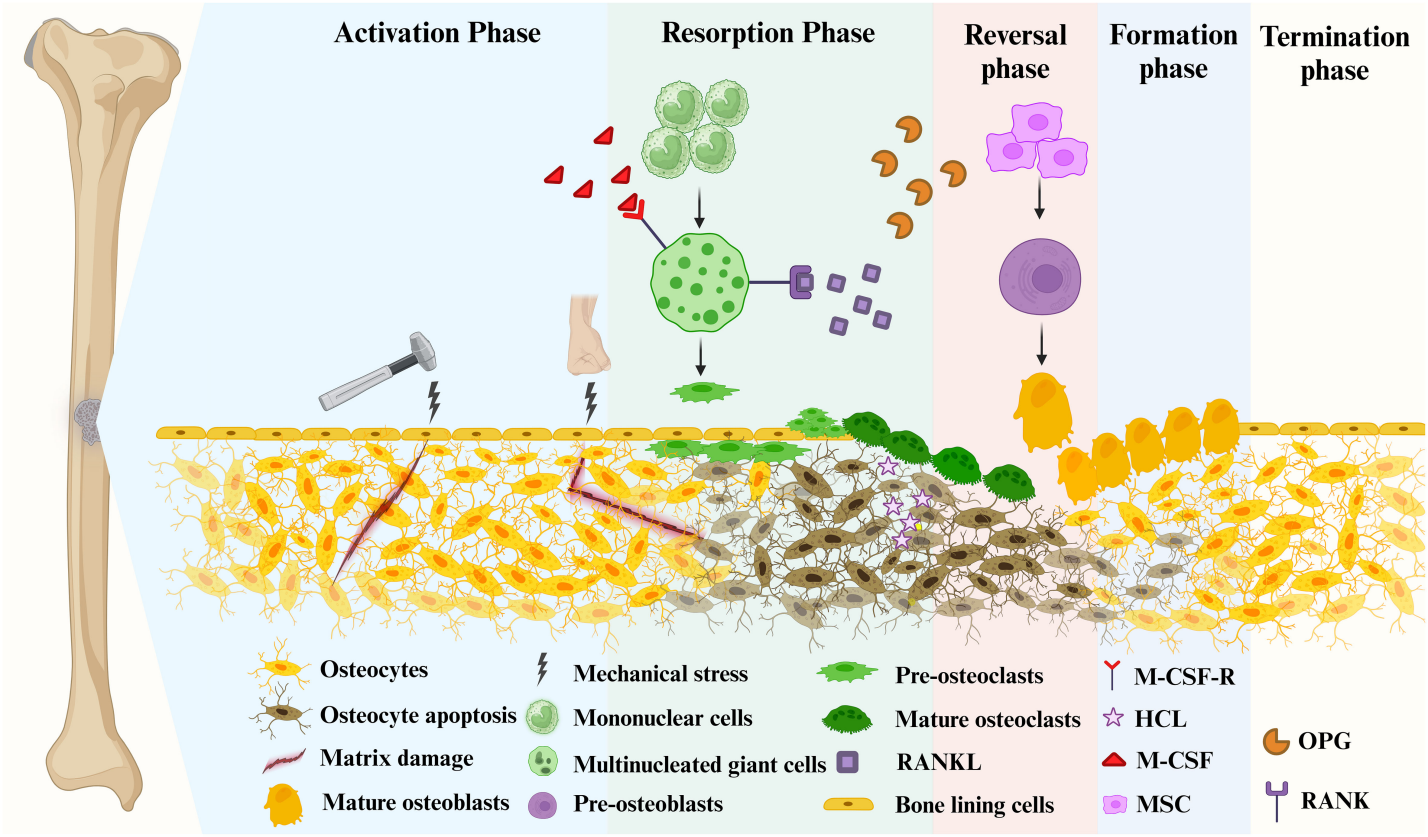
Bone remodeling consists of 5 major distinct but overlapping phases. Mature osteoblasts are generated by the differentiation of mesenchymal stem cells into pre-osteoblasts. Mature osteoclasts arise from multinucleated giant cells differentiated from monocytes stimulated by two important factors, M-CSF and RANKL, followed by differentiation into pre-osteoclasts, and finally into mature osteoclasts. Phase 1 (Activation Phase): Initiating or activating bone remodeling at a specified area. Phase 2 (Resorption Phase): Osteoprogenitors and mesenchymal stem cells are simultaneously recruited while bone resorption occurs. Phase 3 (Reversal Phase): The bone remodeling process shifts from bone resorption to bone formation. Phase 4 (Formation phase): completing bone remodeling and osteoid mineralization. Phase 5 (Termination Phase): New bone replaces old bone to retain bone strength and quality. Maintaining an environment of resting bone surfaces until the next bone remodeling begins (26).

Bone is a dynamic tissue that requires continuous bone remodeling throughout the life span of a human being to maintain its function and normal morphology (22), which is also necessary to maintain calcium levels in the blood. The bone production and resorption processes, which both need communication between different bone cells, are the most crucial components of bone remodeling (**Figure 1**). OB, OC, Bone-lining cells, OYs and their precursor cells in the basic multicellular units (BMUs) promote bone remodeling (23). OBs produce the bone matrix and its subsequent mineralization, and OCs are responsible for bone resorption. The antagonistic activity of OBs and OCs leads to the continued formation and resorption of bone and is the basis for bone remodeling. Briefly, the bone remodeling cycle is tightly regulated by various pathways and signaling molecules, in which new bone replaces old and damaged bone. This process takes 3–6 months to ensure bone integrity and restore bone microdamage by balancing the release of calcium and phosphorus from the host (24). During this process, Bone Remodeling Compartment (BRC) provides a well-defined remodeling region in which OBs and OCs are coupled (25). Bone remodeling is regulated at three levels: directly by OB and OC, indirectly by communication between immune cells and various bone cells, and by the neuroendocrine system.

#### 2.1.1 Activation phase

The remodeling signals that trigger the activation phase include direct mechanical stress to the bone causing structural damage and hormonal effects on the bone, such as estrogen (**Figure 1**) (27, 28). When damage to the bone matrix or limb fixation occurs, it induces apoptosis of OYs, inhibiting TGF-β secretion and recruiting pre-osteoclasts to specific bone sites (27). Bone remodeling occurs in the vascularized closed system bone remodeling region (BRC) and is capped by bone lining cells (29–31). Thus, pre-osteoclasts can be recruited from the bone marrow by passing through a monolayer of bone-lining cells, as well as from capillaries that permeate the BRC (21).

#### 2.1.2 Resorption phase

With the proliferation of OCs in significant numbers, the bone remodeling process progresses to its subsequent phase (**Figure 1**). Pre-osteoclasts differentiate into OCs in response to elevated concentrations of M-CSF and RANKL (31, 32). During this phase, bone resorption becomes predominant. Besides, MSCs and OBs can be recruited into BRC directly from bone marrow or capillaries. Throughout this stage, OC formation and bone resorption persist, while recruited MSCs gradually undergo differentiation into OBs (21). In mechanically induced bone remodeling, OCs adhere to the bone surface through αvβ3 integrins. Furthermore, the actin ring of OCs forms a sealing zone that tightly encircles both the periosteum and the bone surface (33). Hydrogen ions are released into the sealing zones, causing disintegration of the mineralized matrix and production of Howship’s resorption lacunae. Collagenolytic enzymes that function optimally in low pH environments subsequently degrade the collagen-rich bone matrix (34, 35).

#### 2.1.3 Reversal phase

During the reversal phase, bone remodeling process shifts from bone resorption to formation (**Figure 1**). The newly resorbed bone surface provides signals that combine bone resorption with bone production, ensuring no net bone loss. This is achieved through the actions of cells of the OB lineage, which prepare the resorbed bone surface for new bone matrix deposition. These cells first remove the unmineralized collagen matrix on Howship lacunae and then deposit non-collagenous mineralized matrix cement lines to enhance OB attachment (36). Cells in the reversal phase receive or couple signals that facilitate the transition, further highlighting the intricate mechanisms involved in bone remodeling.

#### 2.1.4 Formation phase

Moving from the reversal phase to the Formation phase and performing bone formation in the exact location is very complex (**Figure 1**). The coupling phenomena involved are regulated by multiple mechanisms, including both direct contact (bidirectional cell-anchored EphB4–ephrin–B2 signaling complex) and soluble signals (soluble molecule sphingosine 1-phosphate) (37). The bone mineralization process aims to give the freshly created bone its final shape. Although the exact mechanism of new bone formation is not completely discovered, it is known that OBs produce bone matrix rich in collagen type 1 and control the mineralization of bone-like tissue (38). The collagenous bone matrix forms a new bone surface with various proteins (e.g. proteoglycans, glycosylated proteins, etc.). Hydroxyapatite is integrated to create the newly deposited bone matrix, which is necessary for the bone to take on its final shape (39).

#### 2.1.5 Termination phase

After the replacement of absorbed bone with an equal amount, the remodeling cycle concludes, and the relevant signal is triggered to terminate the remodeling mechanism (**Figure 1**) (40). Mature OBs undergo apoptosis at the end of mineralization. They are ultimately buried in the bone matrix or develop into bone lining cells in preparation for the next bone remodeling. The resting bone surface microenvironment is re-established until the next bone remodeling. After each remodeling cycle in healthy bone remodeling, there is no overall difference in bone mass and strength. In osteoporosis, this process becomes imbalanced, leading to excessive bone resorption compared to bone formation.

### 2.2 Osteoporosis-associated bone cells

As mentioned above, there are various types of cells in bone, including OBs, OCs, OYs, MSCs, and HSCs. Since the underlying cause of osteoporosis is a disorder of bone remodeling, we can assume that the cells participating in bone remodeling are the cells most directly related to OP, namely OBs, OCs and OYs.

#### 2.2.1 Osteoblasts in bone remodeling

Osteoblasts(OBs) are generated from MSCs in the bone marrow, but MSCs can also differentiate into other cells, such as adipocytes and myoblasts (41). The differentiation of OBs proceeds roughly in three parts and is regulated by various molecules. Following MSC differentiation, osteogenic progenitor cells develop into preosteoblasts, mature functioning OBs develop from preosteoblasts (41). Runx-2 and its downstream regulator, Osterix, are two crucial transcription factors that regulate the differentiation of MSCs into OBs in response to external stimuli. Runx-2 will control the differentiation of MSC into preosteoblasts along with its co-activator (42). Then Runx-2 will work with Osterix to further direct the differentiation of preosteoblasts into immature OBs. Critical factors of OB differentiation include PTH, PGE2, IGF-1, Bone morphogenetic protein (BMP), and Wnt proteins (43). Among the multiple pathways that regulate MSC differentiation, the Wnt pathway is the most important and recognized pathway that controls OB formation and is often applied in osteoporosis research (described in detail in 1.3.1). During bone remodeling, OBs produce bone by synthesizing extracellular matrix proteins (the most common type I collagen). After being deposited, the extracellular matrix undergoes mineralization as calcium phosphate accumulates as hydroxyapatite (Ca_10_(PO_4_)_6_(OH)_2_) (44). Moreover, OBs can modulate OC function by regulating their activation or inhibition through the expression of RANKL and M-CSF (25), or NO and OPG, respectively, thus regulating bone mass (45).

#### 2.2.2 Osteoclasts in bone remodeling

OCs originate from monocytes (hematopoietic precursor cells) that differentiate under certain signals into multinucleated cells containing up to 20 nuclei (32). The demineralization of inorganic components and removal of the organic matrix from bone are the two major steps involved in osteoclast-mediated bone resorption. OC defects can result in an imbalance in bone remodeling and pathological conditions such as osteoporosis, bone metastases, and inflammatory bone diseases (46, 47). The M-CSF/M-CSF-R and RANKL/RANK/OPG signaling pathway are vital in regulating OCs. (described in detail in 1.3). Moreover, various transcription factor, such as Purine Rich Box-1 (PU.1), c-Fos, C/EBPα, NFATc1 and MITF, are involved in the regulation of osteoclastogenesis (48–52).

#### 2.2.3 Osteocytes in bone remodeling

The cell type present in bone that is by far the most prevalent is the OY. Up to 25,000 OYs can be found in a cubic millimeter of bone. These cells communicate with one another and the bone surface via tubules (canaliculi), establishing a vast and dense network (33). OYs translate mechanical stimuli exerted on bone into cellular signaling to regulate bone homeostasis (53). SOST, Wnts, Dickkopf-1(Dkk1), Dentin matrix protein 1 (DMP1), and IGF-1 are a few proteins or signaling molecules OYs produce that control OB and OC activities (53–56). Osteoporosis is one of the pathologic disorders that result from an abnormal expression of these molecules.

### 2.3 Critical signaling pathways for bone remodeling

**Figure 2:**
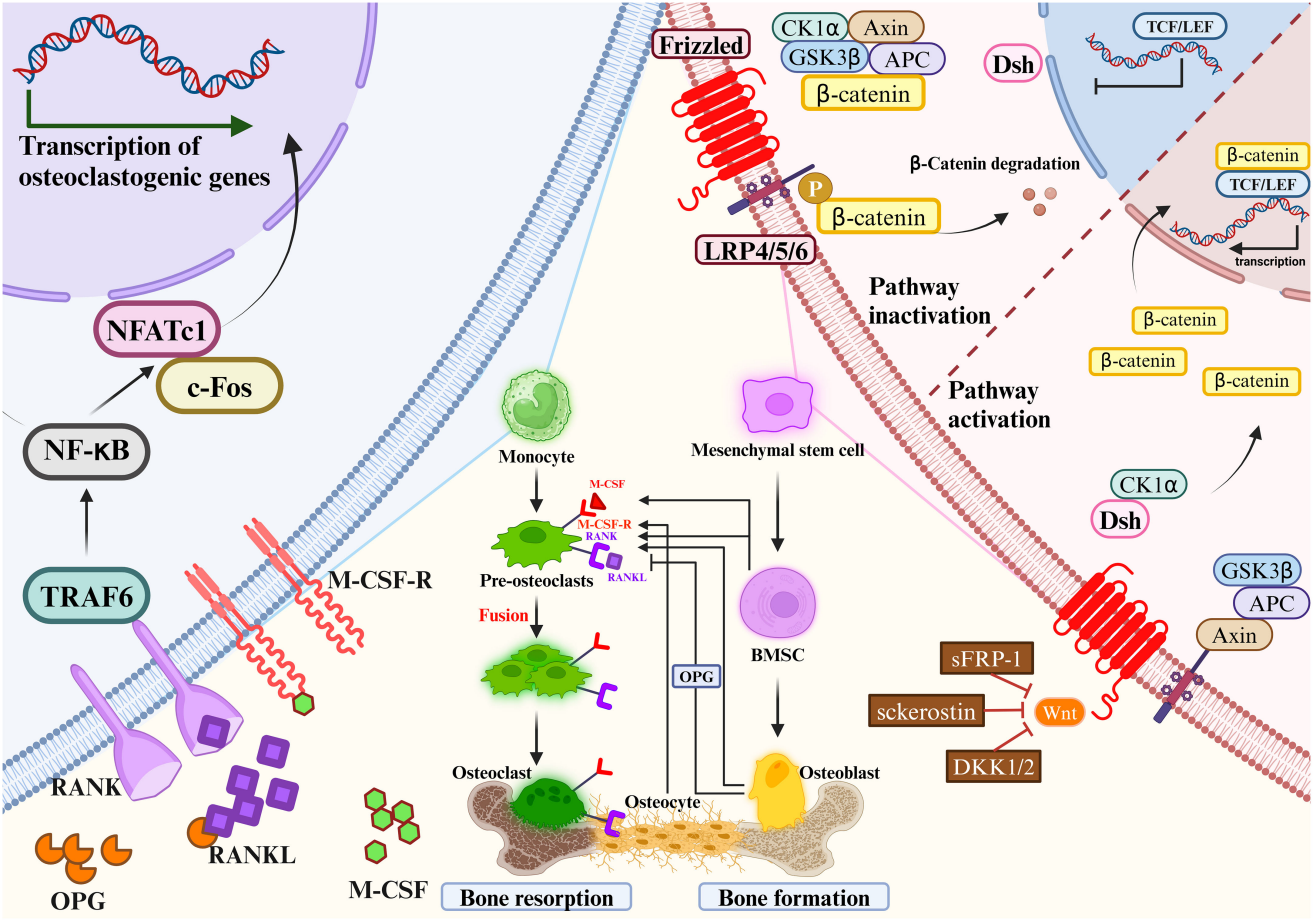
Mesenchymal stem cells gradually differentiate to mature osteoblasts. Monocytes gradually differentiate to mature osteoclasts. Osteoclast precursors express RANK, which binds to the key osteoclastogenic cytokine RANKL.M-CSF binds to its receptor M-CSF-R, the concentration of which directly influences RANKL-mediated OC maturation and differentiation. OPG and RANKL competitively bind to RANK, and therefore OPG inhibits RANKL activity. Mesenchymal stem cells differentiate into bone marrow stromal cells, which then further differentiate into OBs. OBs will differentiate into OYs under the right conditions. OBs and OCs are the main source of RANKL and OPG. The most important signaling pathways in osteoblast-mediated bone formation and osteoclast-mediated bone resorption are the Wnt/β-catenin signaling pathway (left) and the RANKL/RANK/OPG signaling pathway (right), respectively. RANKL/RANK/OPG signaling are essential regulators of bone metabolism. Wnt signaling increases bone mass through a variety of mechanisms, including stem cell renewal, stimulation of preosteoblast replication, induction of osteoblastogenesis, and inhibition of osteoblast and OY apoptosis (left). In the absence of Wnt activation, β-catenin is immobilized by the destruction complex composed of GSK3β, Axin, APC and CK1α. OB activity and quantity are increased when Wnt signaling is activated by the loss of secreted frizzled-related protein 1 (SFRP1), SOST, or a single allele of Dkk1. Wnt ligands stabilize β-catenin by interacting with the LRP5/6 coreceptor through the FZD, thereby sparing β-catenin from phosphorylation and degradation. DSH interacts with the receptor complex and translocates the newly synthesized β-catenin to the nucleus. β-catenin cooperates with TCF transcription factors to promote gene transcription. RANKL/RANK/OPG signaling are essential regulators of bone metabolism (right). At certain concentrations of M-CSF, RANKL binds to RANK and activates intracytoplasmic signaling cascades via the junctional proteins TNF receptor-associated factors (TRAFs). The cytoplasmic region of RANK associates with TRAF6 and transmits RANK stimulation to NF-κB, which activates and translocates NF-κB into the nucleus, increasing the expression of c-Fos. c-Fos activates the expression of NFATc1, initiates osteoclastogenic gene transcription, ultimately inducing the formation of mature OCs. c-Fos, interacting with NFATc1, trigger the transcription of osteoclastogenic genes. Simultaneously, OPG secreted by OBs and OYs competitively binds to RANKL in the form of dimers, reducing the activity of RANKL-RANK and inhibiting OC differentiation, activation, and maturation, and thus bone resorption.

The maintenance of bone homeostasis requires the involvement of multiple signaling pathways. In the process of OCs proliferation and differentiation, common signaling pathways include: RANKL/RANK/OPG signaling pathway, IL-1/TNF-α signaling pathway and MALT1 signaling pathway (57, 58). Common signaling pathways involved in OB proliferation and differentiation include: Wnt/β-catenin signaling pathway, Hedgehog signaling pathway and Notch signaling pathway (59, 60). The most important Wnt/β-catenin signaling pathway in OBs and the RANKL/RANK/OPG signaling pathway in OCs are highlighted in this section (**Figure 2**).

#### 2.3.1 Wnt/β-catenin signaling pathway in osteoblasts

The Wnt/β-catenin signaling pathway regulates bone homeostasis by stimulating osteoblastogenesis and reducing OC differentiation (61). The key mechanism by which Wnt signaling regulates bone remodeling is by stimulating OB development. Importantly, Wnt/β-catenin (i.e., typical) signaling acts to translocate β-catenin to the nucleus (62). Wnt/β-catenin signaling regulates bone development and homeostasis through different mechanisms at each stage of bone remodeling. Wnt signaling increases bone mass through a variety of mechanisms, including stem cell renewal, stimulation of preosteoblast replication, induction of osteoblastogenesis, and inhibition of OB and OY apoptosis (63–65). OB activity and quantity are increased when Wnt signaling is activated by the loss of secreted frizzled-related protein 1 (SFRP1), SOST, or a single allele of Dkk1 (66). β-catenin plays a crucial role in the initial stages of osteoblastogenesis, and its absence redirects the differentiation of mesenchymal precursors toward chondrogenesis (67, 68). In addition, β-catenin regulates osteoclastogenesis by modulating the expression of OPG and RANKL (69, 70). Importantly, activation of Wnt/β-catenin signaling inhibits adipogenesis in MSC precursors, which may be clinically important given the positive correlation between bone marrow adiposity and fractures (62).

Wnt signaling promoted MSC development into OBs and inhibited their development into adipocytes or chondrocytes, such as Wnt10a, Wnt10b and Wnt6 (71). Besides, various Wnts such as Wnt1, Wnt7b, Wnt10b and Wnt16, as well as Frizzled (FZD) receptors such as Fzd7 and Fzd9, have been proven to regulate bone formation (72, 73). Wnt ligands stabilize β-catenin by interacting with the LRP5/6 coreceptor through the FZD, thereby sparing β-catenin from phosphorylation and degradation (74). Then, β-Catenin moves into the nucleus and modulates the osterix and Runx2 expression. These act as key bone-specific transcription factors during osteogenesis. β-catenin regulates multiple stages of OB and OC differentiation and tends to promote bone formation (75). Elevated expression levels of Wnt10a and Wnt10b in the typical β-catenin pathway significantly inhibited adipogenesis and promoted osteoblastogenesis (76, 77). Wnt6 knockdown strengthened osteoblastogenesis and promoted preadipocyte differentiation. Wnt10b stimulates osteoblastogenesis, which accelerates postnatal bone production (78), Wnt7b can stimulate OB differentiation by activating protein kinase Cdelta (PKCdelta) (79), and Wnt3a can activate TAZ through protein phosphatase-1-catalytic subunit alpha (PP1A)-mediated dephosphorylation, which can then drive osteogenic differentiation (80). Moreover, miRNAs are crucial in controlling Wnt signaling. MiR-27a binds to the 3′-untranslated region of the activated protein C (APC) to inhibit OC differentiation and bone resorption (81). Moreover, the Wnt pathway is regulated by the miRNAs. OP’s pathophysiology, stromal mineralization (82, 83), and OB differentiation may be influenced by miR-16–2*, which does so by regulating Runx-2 expression.

#### 2.3.2 RANKL/RANK/OPG signaling pathway and M-CSF/M-CSF-R signaling pathway in osteoclasts

RANKL/RANK/OPG signaling are essential regulators of bone metabolism (25). Dysregulation of this pathway by various factors can result in bone metabolic diseases. RANK, a type I transmembrane protein of the TNF family, is expressed in various cells, including OC precursors, OBs and dendritic cells. RANKL, a type II transmembrane protein and a TNF ligand, is produced by OBs and their precursors, T cells, B cells, and megakaryocytes (84–86). OPG is a soluble glycoprotein, also known as OC inhibitory factor, secreted by MSCs-derived cells such as OBs and BM-MSC (87, 88).

Expression of M-CSF by OBs and bone marrow stromal cells is essential in the differentiation and maturation of OCs (89). As one of the ligands of M-CSF-R, M-CSF plays a key role in the differentiation of OC precursor cells and the maturation of OBs. Xu-Ming Dai et al. demonstrated that M-CSF-R-deficient mice showed decreased parameters of bone strength, abnormal value-added OCs differentiation, decreased bone mineralization, and disturbed bone matrix structure (90). At certain concentrations of M-CSF, RANKL binds to RANK and activates intracytoplasmic signaling cascades via the junctional proteins TNF receptor-associated factors (TRAFs) (91). RANK has three binding sites for TRAF2, TRAF5 and TRAF6, of which TRAF6 is associated with OB differentiation. RANK/TRAF controls OC formation, activation, and survival through a variety of signaling pathways (92, 93).

RANKL binding to RANK activates signaling pathway. The cytoplasmic region of RANK associates with TRAF6 and transmits RANK stimulation to NF-κB (85), which activates and translocates NF-κB into the nucleus, increasing the expression of c-Fos. c-Fos activates the expression of NFATc1, initiates osteoclastogenic gene transcription, ultimately inducing the formation of mature osteoclasts (94, 95). Simultaneously, OPG secreted by OBs and OYs competitively binds to RANKL in the form of dimers, reducing the activity of RANKL-RANK and inhibiting OC differentiation, activation, and maturation, and thus bone resorption (96). The amount of OPG plays an essential role in bone resorption, bone mass and skeletal integrity. Coordination of the RANKL/OPG ratio is crucial to maintain the balance of local bone remodeling. In conclusion, binding RANKL and M-CSF to RANK and M-CSF-R initiates a network of biochemical events that drive osteoclastogenesis. As a result, OPG significantly affects the proliferation, differentiation, and maturation of OBs. If the gene expression of OPG is downregulated, it will affect the pathophysiology of bone resorption, OP and other bone diseases. Therefore, prevention and treatment of inflammatory bone diseases such as osteoporosis is preferred to means of inhibiting RANKL and M-CSF expression and promoting OPG expression, which would prevent clonal amplification of OC while increasing proliferative differentiation of OB.

## 3 Interaction of osteoimmunology with the cytokine IFN-γ

### 3.1 Osteoimmunology: interaction between the skeletal and immune systems

**Figure 3:**
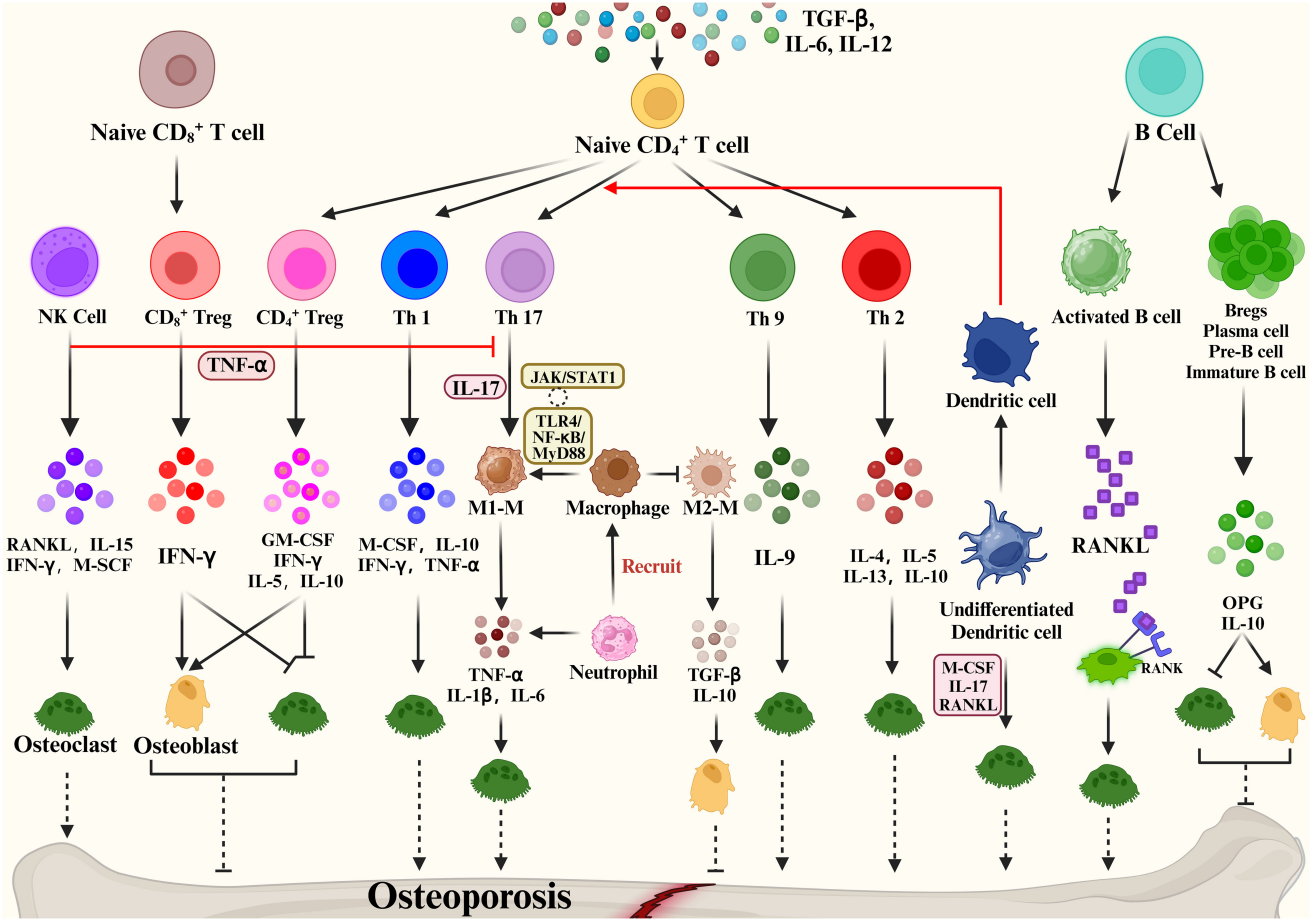
Immune cells mediate bone remodeling and osteoporosis via cytokine production.

The expression of RANKL, RANK, and OPG was observed not only in the skeletal system but also in the immune system (97). Activated T cells, B cells, NK cells, fibroblasts and thymocytes in the immune system can also produce RANKL (98), while dendritic cells also express RANK (99). Besides, the cross-talk between these two systems includes (1) OCs originate from hematopoietic stem and progenitor cells (HSPCs). (2) OB and OC progenitors in the bone marrow coexist with immune cell progenitors and memory cells. (3) The immunomodulatory effect of the osteoclastogenic cytokine RANKL is expressed in both OCs and lymphocytes. (4) Interaction of immune cells and bone remodeling-related cells such as OB and OC in cell differentiation and bone remodeling (100). The microenvironment is the same for both the two systems. The immune system controls OYs by releasing inflammatory agents and associated ligands, which impact bone resorption and formation. Traditionally, osteoporosis is thought to be an imbalance in bone remodeling between OCs and OBs, and a variety of immune cells produce various cytokines that cross-talk with bone to regulate bone metabolism (**Figure 3**).

Some studies show that activated T cells are the major source of RANKL and TNF-α, which cause bone degradation in various pathological and inflammatory situations (101). When IL-12 stimulates the development of naïve CD4 T cells into Th1 cells, IFN-γ, IL-2, lymphotoxin, and TNF-α are generated (102, 103). Of these, IFN-γ is often recognized as inhibiting osteoclastogenesis (104). The Th2 cytokines IL-4, IL-5 and IL-13 inhibit OC formation and bone resorption (105–107).

The cytokine IL-17 produced by Th17 cells has the strongest ability to promote osteoclastogenesis (108). Naive CD4^+^ T cell production of TGF-β and IL-6 affects Th17 cell differentiation and may promote bone resorption. High levels of RANKL are expressed on Th17 cell surface, and RANKL binds to RANK on the OC precursor cell surface, promoting OC precursor cells to OCs and accelerating bone resorption (109). In addition, Th17 produced IL-17 which mediates the secretion of TNF-α, IL-1 and IL-6 by macrophages promotes osteoclastogenesis and inhibits OB activity, promoting bone destruction (110). Based on the important properties of Th17, Th17 cells in peripheral tissues and blood are crucial osteoporosis markers. Another subpopulation of T cells is regulatory T cells (Tregs). Numerous studies have shown that Tregs inhibit OC differentiation and function *in vitro* and vivo (111), thereby reducing the development of inflammatory bone disease, with cytokines produced by regulatory T cells playing a pivotal role. Yong Gil Kim et al. found that Tregs inhibited OC differentiation from PBMCs cytokine-dependently with TGF-β and IL-4 (112). Tregs also inhibited osteoclastogenesis in postmenopausal osteoporosis models by producing cytokines such as IL-10 and IL-5 ultimately reducing bone resorption (113). Briefly, Tregs inhibit the growth and differentiation of OCs in two main ways: (1) Naive CD4^+^ T cells promote the CD4^+^ Tregs differentiation through the expression of TGF-β, which inhibits OC proliferation and differentiation through the production of M-CSF, IFN-γ, IL-5, IL-10. (2) Naive CD8+ T cell-derived CD8^+^ Treg inhibit OC production and activity by increasing IFN-γ expression, which is a negative feedback regulation, and an increase in OCs promotes the value-added of Naive CD8^+^ T cells. Based on the promotion of bone resorption by Th17 and bone formation by Treg, factors that regulate the balance of Th17/Treg cells are now considered as a potential osteoporosis treatment.

In addition, Natural Killer (NK) cells can generate IFN-γ, which indirectly activates T cells and macrophages and releases TNF-α. TNF-α promotes the expression of RANKL and M-CSF by OBs and stromal cells to regulate bone resorption under inflammatory conditions (114). NKT cells can also produce RANKL and M-CSF, which induce osteoclastogenesis and are further regulated by IL-15.

A close bidirectional association exists between B cells and OBs, but this association is less frequent than T cells. Activated B cells can produce cytokines or RANKL involved in bone remodeling. Leena Sapra et al. revealed that Regulatory B Cells (Bregs) could inhibit OC differentiation and thus achieve anti-osteoclastic properties through the production of IL-10 (115). B cells can produce IL-35 to reduce TNF-induced osteoclastogenesis and increase OC apoptosis. By triggering JAK1/STAT1, the activation of TRADD-FADD-caspase 3 is switched from TRADD-TRAF2/RIP1-NF-κB signaling (116). In addition, dendritic cells, as one of the key immune cells in the bone remodeling process, immature DC can be differentiated into OCs in the presence of M-CSF, IL-17 and RANKL. Mature DCs can promote the activation and growth of Th17 cells, thus improving osteoclastogenesis (116). The above evidence demonstrates the involvement of cytokines in bone metabolism and their impact on many bone diseases.

### 3.2 IFN-γ and its signal transduction

IFN-γ is frequently mentioned as an essential cytokine in bone immunology. We know that IFN-γ is significantly associated with bone homeostasis, immune and inflammatory responses, joint disease, bone damage and bone loss (117). The interferon family includes type I IFN, type II IFN and type III IFN. Type I IFN mainly functions as antiviral and antitumor, and there are at least 8 subtypes, including IFN-α, β, κ, τ, δ, ω, ε, and ζ (118). Many cells can produce type I interferons, depending on the cell type and environmental context (119). For example, synoviocytes, phagocytes, fibroblasts, epithelial cells, and dendritic cells all produce IFNβ; plasmacytoid dendritic cells (pDCs) produce IFNα and high levels of IRF7 further promote IFNα production by pDCs (119, 120). Type II interferons, which have only one member, IFN-γ, have immunomodulatory functions with production mainly by immune cells (121), APCs and NK cells in response to immune and inflammatory stimuli. IFN-γ is a multipotent cytokine that activates the innate immune system, promotes the activation of macrophages and mediates the interaction of lymphocytes with other cells (121). It enhances antigen presentation, regulates antiviral and antibacterial immune responses, and regulates the balance between different T cells. It is strongly associated with cell proliferation, differentiation and apoptosis (122). Type III interferons, including IFN-λ1, λ2, λ3 and λ4, are essential regulators of innate anti-fungal immunity.

#### 3.2.1 IFN-γ is involved in immune function

IFN-γ was first discovered in 1965 (123) and is secreted mainly by activated lymphocytes such as CD4 T cells, CD8 T cells, NK cells and γδ T cells; other sources include B cells, and antigen-presenting cells. During the adaptive immune response, Th1 and CTL generate IFN-γ in response to the presentation of antigenic material by MHC molecules (124). During the innate immune response, NK cells produce IFN-γ upon stimulation by IL-12 and IL-18 (125). IFN-γ functions by binding to the IFN-γ receptor (IFNγR), a pre-assembled heterotetramer of IFNγR1 and IFNγR2 subunits associated with JAK1 and JAK2 kinases, respectively. Functionally, IFN-γ can bind to IFNγR1/IFNγR2 to activate STAT pathways and JAK signaling transducers to coordinate various cellular functions (126).

#### 3.2.2 IFN-γ/IFNγR/JAK/STAT pathway

**Figure 4:**
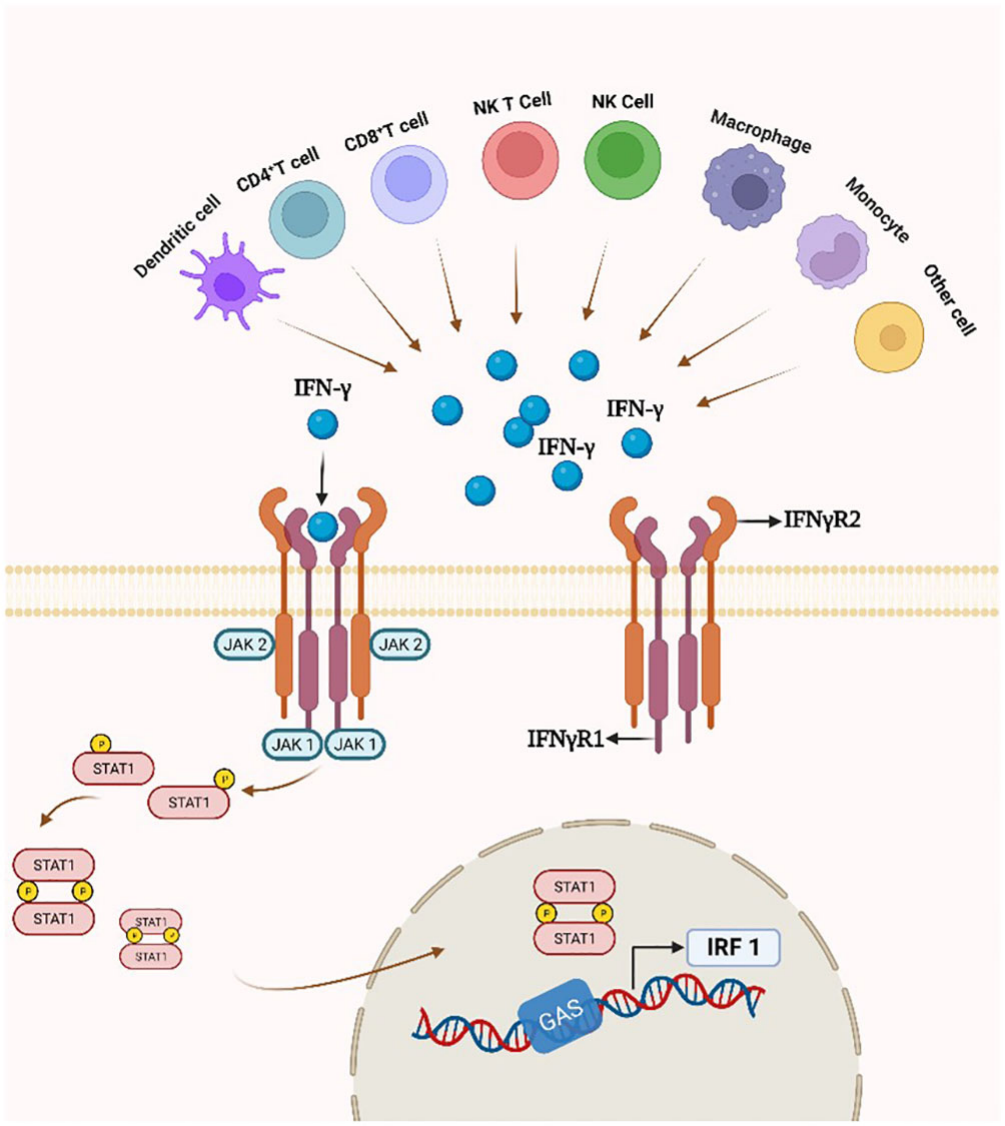
IFN-γ production and the IFN-γ/IFNγR/JAK/STAT pathway. By attaching to a cell-surface receptor made up of two subunits, IFNγR1 and IFNγR2, linked to JAK1 and JAK2, respectively, IFN- γ stimulates the JAK/STAT1 pathway. STAT1 gets phosphorylated, starting the transcription of genes.

In the classical IFN-γ/IFNγR/JAK/STAT pathway, IFN-γ is an antiparallel dimeric peptide, and IFN-γ binds to the cell surface receptor IFNγR1 to induce its dimerization and exposes the binding site with IFNγR2. Upon heterodimerization of IFNγR, the Janus family nonreceptor tyrosine kinases JAK1 and JAK2 are located at the carboxyl terminus of IFNγR1 and IFNγR2, respectively (127). Phosphorylation of the receptor exposes the STAT 1 protein binding site, resulting in endocytosis of the IFN-γ-IFNγR1 complex, transfer of IFNγR1 to the intracellular domain, and movement of JAK2 to IFNγR1. Subsequently, activated JAK1 and JAK2 will phosphorylate the IFNγR1 cytoplasmic structural domain and activate STAT1 protein. Phosphorylated STAT1 homodimer translocates to the nucleus and binds to the downstream target gene promoter GAS site of the IRF-1 gene (**Figure 4**) (128).

## 4 Roles of IFN-γ in OP

**Figure 5:**
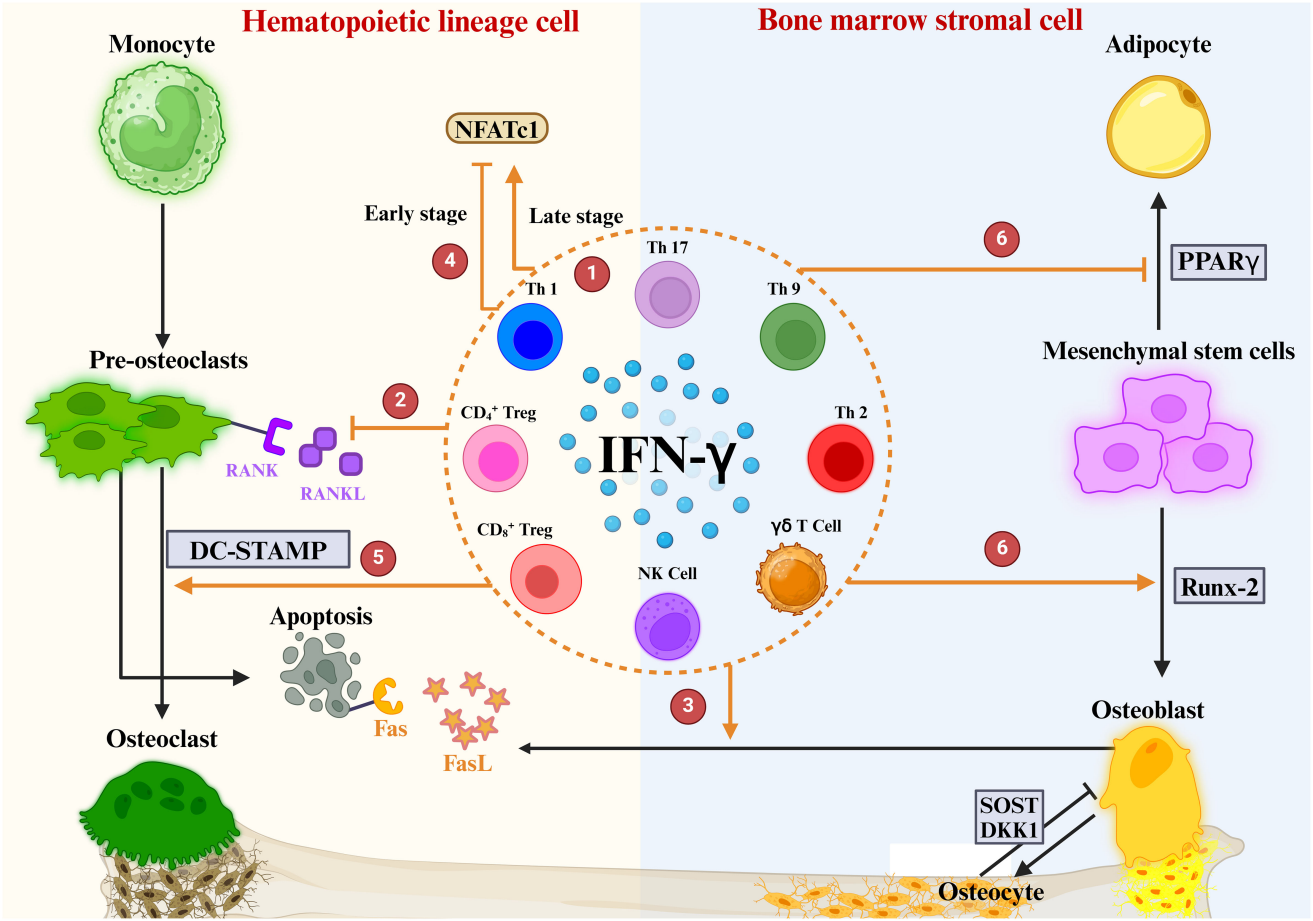
Role of IFN-γ in OBs and OCs. **①** IFN-γ is produced by a variety of immune cells. **②** IFNγ directly inhibits OC differentiation by blocking the RANKL signaling pathway **③** IFN-γ enhances FasL expression in OBs, promotes apoptosis of OC precursors, and ultimately inhibits OC maturation and differentiation. **④** The regulation of NFATc1 downstream of the RANK-RANKL pathway by IFN-γ is highly dependent on the period of OC differentiation. **⑤** IFN-γ mediates DC-STAMP to promote OC maturation. **⑥** IFN-γ upregulates the osteogenic transcription factor Runx2 and downregulates the lipogenic transcription factor PPARγ.

IFN-γ first came to light in osteoimmunology when researchers discovered that it inhibits OC overgrowth and thus has osteoprotective effects (129). Recently, many studies have shown that the connection between IFN-γ and osteoporosis is very complex, and it has osteoprotective and osteodestructive effects (**Table 1**) (130). We explored the possible mechanisms of IFN-γ-mediated osteoporosis from three aspects: the effect of IFN-γ on OCs, the effect of IFN-γ on OBs and the effect of IFN-γ on bone mass (**Figure 5**).

**Table 1:** Studies reported that IFN-γ mediates the occurrence and development of osteoporosis by affecting bone remodeling.

Author/Year	Diseases/Animal modal/Cell model	Main function
Fawaz Y Azizieh et al./2019 (131)	Healthy, osteopenic and osteoporotic postmenopausal women.	In postmenopausal women, osteoporosis serum levels of IFN-γ, IL-12p70, IL-33, IFN-α2, and MCP-1 were significantly elevated.
Bolaji Lilian Ilesanmi-Oyelere et al./2019 (132)	86 menopausal women were categorized into three groups: healthy, osteopenic and osteoporotic.	The levels of cytokines (IFN-γ, IFNα2, IL-33, IL-12p70, IL-12p70) and MCP-1 in plasma were significantly higher in the osteoporosis group.
V Breuil et al./2010 (133)	26 postmenopausal women with osteoporosis and 24 healthy women.	Postmenopausal osteoporotic women exhibit significantly reduced IFN-γ secretion by CD4+ T lymphocytes.
Wei Zhang et al./2022 (134)	Healthy, osteopenic and osteoporotic elderly male.	Elderly male patients with osteoporosis had higher expression of RANKL/OPG, TNF-α, IL-6, and lower expression of IFN-γ and IL-10.
Cong Peng et al./2022 (135)	20 elderly osteoporotic patients and 20 non-osteoporotic healthy individuals.	IFN-γ levels were significantly lower in elderly patients with osteoporosis.
Tiago Azenha Rama et al./2023 (136)	Systemic Mastocytosis patients (n=120) were categorized according to their bone status: healthy bone, osteoporosis and diffuse osteosclerosis.	In osteoporosis patients, there is a notable elevation in serum baseline levels of tryptase, IFN-γ, IL-1β, and IL-6.
Roba M Talaat et al./2015 (137)	20 osteopenic and 20 osteoporotic patients treated with bisphosphonates.	In treated osteoporosis patients, there was a significant decrease in plasma levels of IFN-γ, IL-17, IL-23, and IL-6.
Simone Cenci et al./2003 (138)	IFN-γR knock-out mice and their WT littermates; ovariectomized and sham-operated mice.	In estrogen-deficient conditions, the induction of CIITA by IFN-γ escalates antigen-presenting cell (APC) activity and T-cell proliferation. This process contributes to the expansion of the T-cell pool, heightened TNF production, and the *in vivo* occurrence of TNF-induced bone loss.
Yuhao Gao et al./2007 (139)	IFN-γR knock-out mice and their WT littermates; ovariectomized and sham-operated mice.	The activation and proliferation of antigen-dependent T-cells driven by IFN-γ, along with elevated concentrations of RANKL and TNF-α, foster osteoclastogenesis, ultimately resulting in a net loss of bone tissue.
L Wang et al./2015 (140)	Ovariectomy-induced osteoporotic mice and sham-operated mice; FASL-null mice and their littermates; IFN-γ knockout mice and their littermates.	Administration of IFN-γ to OVX-induced osteoporotic mice ameliorated the osteoporotic phenotype and increased the expression of FASL in osteoblasts, promoting osteoclast apoptosis and inhibiting bone resorption.
Gustavo Duque et al./2011 (141)	IFN-γR knock-out mice and their littermates; OVX-induced osteoporotic mice and sham-operated mice.	Compared with IFNγR1(+/+) mice, IFNγR1(-/-) mice had reduced bone mass and bone mineral density and exhibited significant osteoporotic features. In addition, administration of IFN-γ to OVX female mice significantly improved bone remodeling, bone mechanical properties, bone microarchitecture, and bone mass, and ameliorated osteoporosis in OVX mice.
Miyuki Tsumura et al./2022 (142)	Patients with autosomal dominant (AD) IFN-γR1 deficiency.	Patients with AD IFN-γR1 deficiency have an increased proportion of GM colonies differentiating into osteoclasts in response to RANKL and M-CSF. IFN-γ concentration-dependent inhibition of osteoclast formation is impaired.
Elena V Shashkova et al./2016 (143)	IFN-γR knock-out mice and their littermates.	TcREG inhibits actin reorganization and osteoclast attachment to the bone matrix by blocking the αvβ3 signaling of IFN-γ, which ultimately inhibits bone resorption.
Masatsugu Komagamine et al./2023 (144)	Collagen-induced arthritis (CIA) mice	JAK inhibitors reduce the number of osteoclasts and ameliorate systemic bone loss. JAK inhibitors attenuate the inhibitory effect of Th1 cells on osteoclastogenesis by inhibiting IFN-γ signaling in osteoclast precursor cells.
Grant S Schulert et al./2021 (145)	Bulk RNA-seq was performed on peripheral blood mononuclear cells and single cell (sc) RNA-seq analysis was performed on bone marrow macrophages from 26 Systemic juvenile idiopathic arthritis (SJIA) patients.	The positive regulator of IFN-γ signaling, TRIM8, was upregulated in SJIA patients. In addition, scRNA sequence analysis of BMM indicated that the IFN-γ response pathway was upregulated.
Edit Nagy et al./2017 (146)	Osteoclast precursor	Treatment of osteoclast precursors with IFN-γ decreased mRNA expression levels of TRAP, Cathepsin K, RANK, and TRAF6. Addition of neutralizing anti-IFN-γ antibody abolished these effects.
S Kamolmatyakul et al./2001 (147)	MOCP-5 and wild-type mouse bone marrow co-culture systems.	IFN-γ downregulated Cathepsin K mRNA expression in a time- and dose-dependent manner, and IFN-γ inhibited osteoclast formation only at early stages of osteoclast differentiation.
Haiyan Li et al./2014 (148)	Osteoclasts and T cells.	Activated T cells exert immunosuppressive functions by inducing IDO expression in OCs via IFN-γ.
Haruka Kohara et al./2011 (149)	Bone marrow macrophages.	IFN-γ indirectly regulates bone resorption and plays an osteoprotective role by inhibiting TNF-α stimulation-induced osteoclastogenesis and accelerating Fas/FasL signaling-mediated apoptosis.
Jingyi Tan et al./2021 (150)	Bone marrow macrophages.	IFN-γ significantly enhanced the upregulation of mRNA expression involving c-Fos, NFATc1, Ctsk, MMP-9, and TRAP in bone marrow macrophages, thereby promoting osteoclast differentiation.
Jiajia Xu et al./2016 (151)	Bone marrow mesenchymal stem cell	IL-12 and IL-23 indirectly inhibit BMMSC differentiation by increasing IFN-γ.
Zhongxiu Wang et al./2018 (152)	Primary osteoblasts	Higher ALP activity was observed in IL-17 and IFN-γ-treated primary osteoblasts.

### 4.1 Role of IFN-γ in mesenchymal stem cells

Mesenchymal stem cells (MSCs), a type of pluripotent progenitor cells, possess multifunctional properties such as tissue regeneration and self-renewal (153). They exhibit the capacity to differentiate into various cell lineages, including OBs, chondrocytes, and adipocytes (154). IFN-γ, a prevalent pro-inflammatory cytokine, has garnered significant attention for its immunosuppressive effects on MSCs (155). T cell-secreted IFN-γ binds to receptors located on MSC membranes, thereby modulating the production of immunosuppressive molecules, such as HGF, TGF-β, and IDO. These molecules inhibit T cell proliferation and activation, augment negative T cell signaling, induce a shift in T cell phenotype from pro-inflammatory to anti-inflammatory, and engage with antigen-presenting cells (155, 156). Moreover, both *in vitro* and *in vivo* investigations have demonstrated the pivotal role of IFN-γ stimulation in directing MSCs toward the OB lineage, with the exogenous addition of IFN-γ further expediting this process (157). The gradual transition of MSCs into mature osteoblasts is governed by a multitude of factors. In a study by Cheng Li et al., stimulation of MSCs with IFN-γ and TNF-α was found to modulate the paracrine effects of MSCs, resulting in a significant elevation in the secretion levels of IL-6, HGF, VEGF, and TGF-β, thereby promoting OB differentiation (158). Additionally, it was discovered that certain IFNγ-induced genes exhibited upregulation during the initial stage of MSC differentiation into OBs (157). Moreover, a transient yet robust surge in IFNγ expression followed hMSC-induced differentiation into OBs (157). Inhibition of autocrine secretion of IFNγ by MSC suppressed the early stages of MSC differentiation into OBs *in vitro (* 157). These findings strongly suggest that IFNγ likely holds significance in guiding MSC differentiation toward mature osteoblasts.

Animal experimentation revealed that IFNγR1-/- mice exhibited reduced BMD, alongside diminished differentiation capacity of their MSCs into osteoblasts (157). Additionally, Rifa et al. demonstrated that T-cell cytokines, including IFN-γ, modulate MSC differentiation and mineralization through the regulation of bone morphogenetic protein 2 (BMP-2) expression (159). Gustavo Duque et al. cell experiments showed a rapid increase in IFNγ production after MSC induced differentiation into OBs. Blocking IFNγ autocrine secretion in MSCs markedly hindered MSC differentiation into OBs, concomitantly reducing expression of the osteogenic transcription factor Runx2 (157). However, the exogenous addition of IFNγ caused MSC to differentiate into OBs in a dose-dependent manner, as well as induced a high level of Runx2 expression during the early stages of differentiation (157). IFN-γ inhibited the differentiation of MSCs into adipocytes through upregulation of the osteogenic transcription factor Runx-2 and downregulation of the lipogenic transcription factor PPARγ (160). Similarly, MSCs derived from IFN-γ receptor-deficient mice exhibited lower osteogenic differentiation capacity due to reduced expression of Runx-2 (141). These findings underscore the essential role of IFN-γ in both *in vitro* osteogenic differentiation of MSCs and *in vivo* maintenance of BMD, suggesting its potential significance in differentiation and bone formation *in vivo*.

### 4.2 Role of IFN-γ in monocytes

Osteoclast precursors are derived from HSCs through monocyte/macrophage cell lines (161–163). F Arai et al. found that M-CSF and RANKL-stimulated OC precursors have the dual potential to differentiate into mature osteoclasts and macrophages (164). In addition, Li et al. found that BMMs have the potential to differentiate not only into OCs but also into dendritic cells (165). These observations suggest that OC precursors have the potential to differentiate into multiple cell types. Upon stimulation by OC differentiation factors such as RANKL, OPG, and ODF, OC precursors commit to the fate of differentiating into OCs and lose their multipotential differentiation ability. As early as 1984, JB Weinberg et al. showed that IFN-γ induces the formation of multinucleated macrophages by mediating the process of monocyte fusion and activates monocytes through various pathways (166). However, IFN-γ only inhibited the early process of BMMs differentiation toward OCs, but not the subsequent differentiation process after commitment (167). In addition, the accessory protein FcRγ chain enhances RANKL signaling by expanding calcium influx required for NFATc1 activation, ultimately promoting osteoclastogenesis (168, 169). Notably, Bettina Groetsch et al. describe a possible structural and functional cooperation between IFNγR and FcRγ on dendritic cells and macrophages (168). Differentiation of monocytes into OCs is influenced by both FcγR and IFNγR signaling and is highly dependent on the stage of differentiation (170). CTSK expression is required for normal bone development, and its lack of expression results in impaired osteoclast-mediated bone resorption (34, 171, 172). Manhui Pang et al. found that IFN-γ inhibited RANKL-induced monocyte differentiation to OCs by stimulating the expression of CTSK mRNA and inhibiting RANKL stimulation of CTSK mRNA and protein (173). Furthermore, IFN-γ triggers rapid TRAF6 degradation in BMM via activation of the classical JAK-STAT1 pathway, consequently robustly inhibiting downstream transcription factors NF-κB and JNK activation (10). Conversely, overexpression of TRAF6 in BMM would counteract the inhibitory effect on OC precursor production (10).

### 4.3 The role of IFN-γ in osteoclasts

#### 4.3.1 *In vivo* studies

K Klaushofer et al. reported that mice treated with IFN-γ showed reduced numbers of OCs (174). Similarly, patients with IFN-γ receptor deficiency have increased OC differentiation (142). IFN-γ significantly mitigated bone loss and OC formation while reducing TNF-α expression and promoting bone formation (175). Furthermore, De Klerck et al. showcased the inhibitory impact of IFN-γ on OC formation in collagen-induced arthritis (CIA) via an IFN-γR knock-out mouse model (176). Arthritis occurred faster and bone destruction was more severe in IFN-γR knock-out mice compared to wild-type mice. Tartrate-resistant acid phosphatase (TRAP or TRAPase) osteoclast-like cells appeared early in the synovial fluid of the joints. This experiment demonstrated a noteworthy elevation in osteoclastogenesis in IFN-γR knock-out mice (176), and this trend was associated with cysteine aspartate protease-1 (caspase-1) activation and IL-1β hydrolysis maturation. Thus, IFN-γ inhibits OC formation *in vivo*.

In addition, IFN-γ also exhibited bone resorptive effects. Yuhao Gao et al. illustrate that the administration of IFN-γ in T-cell-deficient mice does not elicit abnormalities in bone metabolism. However, bone mass experiences a significant reduction in both normal mice and nude mice reconstituted with T cells (139). It is hypothesized that the induction of MHC-II-like molecules expression by IFN-γ may trigger T cells to release RANKL and TNF-α, thereby promoting osteoclastogenesis. Hence, the bone-resorbing impact of IFN-γ necessitates the presence of T cells. Ferrari et al. proposed that *in vivo*, IFN-γ exerts both direct anti-osteoclastogenic effects and indirect pro-osteoclastogenic effects mediated through T cells. Under normal physiological conditions, IFN-γ inhibits osteoclastogenesis, while T cells increase IFN-γ production under estrogen deficiency, inflammation, and infection, with the overall effect of activating OCs to increase bone loss (177). Yuhao Gao et al. concluded that in an experimental model of postmenopausal osteoporosis, the net balance between direct anti-resorptive and indirect pro-resorptive effects of IFN-γ was biased toward osteoclastogenesis and bone resorption (139), which resulted from the upregulation of antigen presentation capacity. During this process, crucial osteoclastogenic factors such as RANKL and TNF-α secretion are heightened, leading to T cell activation and proliferation. This bone resorption-promoting activity overcomes the inhibitory effect of IFN-γ on OC precursors, resulting in net bone loss. In summary, the effects of IFN-γ on OC formation are bidirectional, but the conclusions and induction pathways are unclear and inconsistent. Considering the differences in materials, methods and doses of IFN-γ used by different teams, this conclusion should be interpreted more cautiously and needs to be further demonstrated by well-designed experiments.

#### 4.3.2 *In vitro* study: IFN-γ inhibits osteoclast-mediated bone resorption

Early *in vitro* studies suggested that IFN-γ inhibits osteoclast-mediated bone resorption either by inhibiting OC proliferation (174) or by inhibiting OC precursor fusion (178), rather than directly inhibiting mature osteoclast-mediated bone resorption. This view is consistent with that of *in vivo* studies.

Numerous studies have shown that IFNγ directly inhibits OC differentiation by blocking the RANKL signaling pathway (179). Generally, RANKL activates TRAF6, which further activates NF-κB and JNK pathways, while promoting the expression of C-Fos. TRAF6 and C-Fos are required for OC formation (180). H Takayanagi et al. showed that IFN-γ plays a central role in inhibiting OC formation by RANKL-induced T cells (10). TRAF6 is a crucial transcription factor in the RANKL signaling pathway, and IFN-γ accelerates TRAF6 protein degradation by activating the ubiquitin-proteasome system, thereby inhibiting OC formation and maturation (129).

Su Yang et al. revealed that IFN-γ could induce the expression of peroxides in OC, leading to OC precursors apoptosis and inhibition of OC activity (181). Willis Huang et al. found that the pre-treatment of OC precursors with RANKL counteracted the inhibitory effect of IFN-γ on terminal OC differentiation (182). In another study, S. Kamolmatyakul et al. demonstrated that IFN-γ reduces histone K production and specifically inhibits OC formation only during the early stages of OC differentiation. IFN-γ downregulated cathepsin K mRNA in a time-dependent and dose-dependent manner (147). The effect of IFN-γ on OC bone resorption might be mediated by its effect on early OC formation and gene expression in mature OCs. In summary, IFN-γ exerted a dose-dependent inhibitory effect on the monocyte differentiation to OCs. Additionally, its inhibitory effect on osteoclastogenesis varied with time and declined as OCs matured.

#### 4.3.3 *In vitro* study: IFN-γ promotes osteoclast-mediated bone resorption

In addition to its inhibitory effect on OC differentiation, there is growing evidence that IFN-γ also promotes osteoclastogenesis, thereby increasing bone resorption. P.R. Madyastha et al. discovered that the addition of IFN-γ increased OC production in peripheral blood cells of patients with malignant osteoporosis (183). Another study found that OC precursors pre-exposed to RANKL were resistant to the inhibitory effect of IFN-γ (182). Consequently, there’s a hypothesis suggesting that the direct anti-osteoclastogenic activity of IFN-γ gradually weakens *in vivo* with increasing RANKL concentrations. Moreover, considering that activated T cells serve as the primary source of IFN-γ, a robust amplification loop might be established to perpetuate antigen presentation and sustain T cell activation, thereby maintaining the inflammatory environment.

During the early stages of osteoclastogenesis, IFN-γ processing is pivotal as it predominantly restrains cell growth and activation via ROS and downstream MAPK signaling pathways (170, 184). The regulation of FcγR and IFNγR functions during OC differentiation involves different membrane localization (170). The MAPK downstream signaling pathway is necessary for the observed divergence in IFNγR signaling (170, 184). IFN-γ prevents OC differentiation and activation only by stimulating early pro-OCs, whereas premature stimulation of pro-osteoclasts by IFN-γ even increases the number and resorptive activity of multinucleated OCs *in vitro (* 170). At low RANKL concentrations in the early stages, IFN-γ significantly induced BMM activation and promoted the maturation of OC precursors. However, exposure to high RANKL concentrations in the early phase will result in BMM resistance to IFN-γ, leading to decreased osteoclastogenesis (150, 182). In addition, IFN-γ downregulates NFATc1 to inhibit OC formation during early differentiation but upregulates NFATc1 to increase OC incorporation during late differentiation (185). Hence, the varied impact of IFN-γ on OCs may depend on their maturation stage, and either too early or too late stimulation of OC precursor differentiation by IFN-γ may enhance OC production.

### 4.4 Role of IFN-γ in osteoblasts

In an earlier study, C Ruiz et al. examined the expression of IFN-γ cytokines in human OBs by immunocytochemistry and flow cytometry, which showed that IFN-γ significantly increased the fluorescence intensity of the cytokines (186), so we can know that IFN-γ is significantly expressed in OBs. Apalset et al. studied serum samples and BMD in 5312 subjects and found that the IFN-γ-mediated inflammatory marker (neochrome and kynurenine/tryptophan) content ratio (KTR) was negatively correlated with BMD (187). The lateral response shows that the main role of IFN-γ in the inflammatory state is bone resorption *in vivo*. IFN-γ not only has an effect on OCs but is also associated with OBs.

IFN-γ was found to inhibit OB proliferation and stimulate ALP activity in the early stages. Additionally, IFN-γ has increased STAT1 expression in both humans and mice via a mechanism involving PKR (188). It has been demonstrated that STAT1 plays a crucial role in inhibiting OB differentiation, and is essential for IFN-γ-mediated bone growth inhibition. However, without STAT1, OB differentiation and ALP activity are increased (189). Zha et al. showed that IFN-γ reduced ALP secretion during early differentiation and decreased the expression of Runx-2, a major transcription factor for OB differentiation (190). The results are similar to those of S.J. Gilbert, who showed that IFN-γ significantly reduced the expression of ALP and Col1a1 genes in human and mouse OBs, which supports the action of IFN-γ as an OB differentiation inhibitor (188). Moreover, IFN-γ and TNF-α work together to cause apoptosis in OB by upregulating mitochondrial cytochrome c release or nitric oxide generation, decreasing B-cell lymphoma 2 expressions, and activating caspases (191).

In contrast, many studies have shown that IFN-γ promotes OB formation by upregulating key osteogenic factors, including Runx-2, Osterix, ALP and Osteocalcin (OCN). Gustavo Duque et al. found that IFN-γ receptor-deficient mice exhibit reduced bone mass. There were remarkable changes in cortical and trabecular structural parameters which characterize the osteoporotic phenotype (141). Runx-2 and ALP also decreased significantly compared with the control group, demonstrating that IFN-γ positively regulates the formation of OBs (141). MSCs can differentiate into various cells, including OBs, while several factors regulate OY differentiation. IFN-γ production by MSCs plays a vital role in osteogenic differentiation (157). IFN-γ inhibited the differentiation of MSCs into adipocytes through upregulation of the osteogenic transcription factor Runx-2 and downregulation of the lipogenic transcription factor PPARγ. In contrast, MSCs derived from IFN-γ receptor-deficient mice exhibited lower osteogenic differentiation capacity due to reduced expression of Runx-2 (141). Under normal physiological conditions, OBs secrete FASL (FAS ligand) to induce apoptosis in mature OCs, which is an essential process for maintaining bone mass. L Wang et al. showed that systemic administration of IFN-γ-neutralizing antibodies to modulate pro-inflammatory responses was shown to rescue the reduced FASL expression in OB lineage cells and ultimately alleviate osteoporotic symptoms in ovariectomized (OVX) mice (140). In summary, the effects of IFN-γ on OBs appear similar to those of IFN-γ on OCs, with both producing bidirectional effects. IFN-γ regulates OBs in a stage-dependent manner, with associated proteins and COL1A and BSP decreasing in the early phase and, conversely, mineralizing proteins ALP and OCN being upregulated in the late phase. IFN-γ is a negative regulator of OB differentiation and bone formation in the early stages and changes to a positive regulator in the later stages. However, the effects of IFN-γ during OB differentiation have not been fully studied so far, and the exact process needs to continue to be investigated.

### 4.5 Effects of IFN-γ on bone mass

Studies have shown conflicting results regarding the effect of IFN-γ on bone mass. On one hand, IFN-γ treatment has been shown to reduce spinal bone mass in mice (139), resulting in decreased bone formation and increased bone resorption, ultimately leading to a net loss of bone mass (192). On the other hand, knock-out IFN-γ gene and knock-out IFN-γ receptor gene mice have been shown to exhibit lower bone mass (141). In 3.1 and 3.2, it is known that IFN-γ can act as both a pro- and anti-resorptive cytokine. IFN-γ can directly target OC precursors, thereby inhibiting OC differentiation. However, premature or late stimulation of OC precursors by IFNγ increases the number and resorptive activity of multinucleated OCs *in vitro (* 170). Moreover, it stimulates the activation of T cells and promotes the release of the osteoclast-producing cytokines TNF-α and RANKL, indirectly encouraging bone resorption.

The net effect of IFN-γ on bone is highly concentration and dose dependent. *In vitro* investigations have revealed that 1 ng/ml of IFN-γ significantly impedes BMM differentiation into OCs, while complete inhibition of OC formation is observed at 100 ng/ml, with this inhibitory effect persisting even at a higher dose of 500 ng/ml (139, 174). Conversely, Jingyi Tan et al. reported that the addition of 0.02 ng/ml and 0.2 ng/ml of IFN-γ dose-dependently stimulated BMM differentiation into OCs (150). Similarly, the modulation of OB differentiation by IFN-γ also exhibited dose dependence. *In vitro* experiments demonstrated that IFN-γ dose-dependently (0, 10, 100 ng/ml) augmented OB differentiation and calcium deposition in OB cultures by inducing pathways involving Runx2, Osterix, Alp, and Bglap (193). The net effect of IFN-γ on bone showed similar results in *in vivo* studies. Subcutaneous injection of 9 µg of IFN-γ in rats (three times per week for three weeks) significantly reduced the number of OCs and increased bone formation (194). Likewise, Gustavo Duque et al. demonstrated that administering 2000 and 10,000 units of IFN-γ three times weekly for six weeks to female mice in both sham-operated and OVX groups significantly improved bone mass and trabecular microarchitecture, while alleviating osteoporotic symptoms in OVX mice (141). In contrast, short-term injection of high doses of IFN-γ promoted OC formation and aggravated osteoporosis. Mice receiving intramuscular injections of 100 μg IFN-γ (once weekly for three consecutive weeks) in both sham-operated and OVX groups exhibited varying degrees of osteoporosis and increased bone resorption (195). Similarly, mice were injected intraperitoneally with 1 × 10^6^IU/kg of IFN-γ twice a week for three consecutive weeks, and the net effect of IFN-γ was to stimulate bone resorption and bone loss (139).

It is known that activated T cells promote osteoclastogenesis by expressing RANKL (10). In addition, activated T cells also inhibit osteoclastogenesis through IFN-γ production (196). The balance between the roles of RANKL and IFN-γ is an important regulatory mechanism for osteoclast-mediated bone resorption (196). *In vitro* studies have shown that after preferential exposure of OC precursors to RANKL, the IFN-γ ability to inhibit osteoclastogenesis is significantly reduced (197). In contrast, if OC precursors were preferentially exposed to IFN-γ, or to both IFN-γ and RANKL, osteoclastogenesis was effectively inhibited (198). This is because IFN-γ inhibits osteoclastogenesis by suppressing RANKL-induced activation of NFATc1 and JNK signaling pathways in OC precursors (198). In addition, E. R. Ayon Har et al. observed that IFN-γ supplementation at 0 hours *in vitro* inhibited RANKL-induced osteoclast (OC) formation, whereas this inhibitory effect was attenuated when IFN-γ was supplemented at 48 hours (199). This suggests that the regulation of OC formation by IFN-γ is related to the stage of OC differentiation. We conclude, “The net effect of IFN-γ on bone is closely related to the circumstances, dose, concentration and stage of OC differentiation.” In general, IFN-γ has a direct inhibitory effect on OCs and an indirect promotional effect on OCs. In the case of postmenopausal osteoporosis, infection and inflammation, bone resorption is greater than bone remodeling and net bone mass increases.

## 5 IFN-γ in the treatment of osteoporosis

Osteoporosis varies depending on the specific type of etiology. Primary osteoporosis is caused by endocrine disorders, systemic aging and abnormal bone metabolism in adolescents (200). Secondary osteoporosis is caused by endocrine diseases, autoimmune diseases, digestive system diseases, liver dysfunction, organ transplantation, etc. The inducing factors of these diseases will eventually cause chronic inflammation, decreased nutrient absorption, organ failure, abnormal calcium metabolism, and abnormal regulation of bone metabolism. In addition, drugs such as glucocorticoids, heparin, methotrexate and cyclosporine can also cause secondary osteoporosis (201, 202).

### 5.1 Crosstalk between IFN-γ and postmenopausal osteoporosis (PMOP)

Postmenopausal osteoporosis is a frequent form of primary osteoporosis that is associated with sex hormone levels. The effects of estrogen and androgens on bone have been extensively studied (203), and in women who have undergone menopause, the cessation of ovarian function results in decreased estrogen levels, leading to increased bone turnover and enhanced bone resorption, which is a major contributor to primary osteoporosis (204). In OBs, OCs, and OYs, estrogen receptor (ER) are highly expressed and have protective effects on bone. Through binding to ERs, estrogen regulates the expression of genes that code for proteins, including IL-1, IGF-1, and TGF, and inhibits RANKL (205), thereby preventing OC formation and bone resorption. Alterations in immune status in postmenopausal women can also indirectly contribute to persistent bone destruction, with various immune cells interacting with OBs and OCs via cytokines (IL-6, IL-7, IFN-γ, TNF-α and RANKL) (206).

A controlled study by V Breuil et al. demonstrated that women with postmenopausal osteoporosis (PMOP) had lower levels of IFN-γ secretion in immune cells compared to healthy women, suggesting a potential role for IFN-γ in the pathophysiology of PMOP (133). Similarly, Jing Zhang et al. found significantly lower serum concentrations of IFN-γ in PMOP women, indicating that targeted treatment with IFN-γ may benefit this patient population and confirm the involvement of IFN-γ in inhibiting bone resorption and the pathogenesis of PMOP (207). In contrast, S X Zheng et al. showed no significant differences in IFN-γ concentrations in healthy or PMOP women (208).

In animal experiments, Gustavo Duque et al. demonstrated that injection of IFN-γ significantly enhances bone mass, microarchitecture, bone mechanical properties, and bone formation to resorption ratio in both wild-type and OVX mice. Moreover, IFN-γ treatment was found to be more effective in treating osteoporosis in OVX mice (141). This finding highlights the significant physiological role of IFN-γ signaling as a potential therapeutic target for osteoporosis. In an IFN-γ-mediated immunomodulatory approach, Hao Shen et al. demonstrated that WR exercise prevented bone loss in OVX mice (209). Activated CD8 T lymphocytes restored IFN-γ expression after WR exercise, which prevented OC formation and restored bone loss via NF-κB and MAPK pathways. Consistently, L Wang et al. showed that IFN-γ administration ameliorated OVX-induced osteoporosis and restored FASL expression in OBs (140). OBs induced OC apoptosis via FAS ligand (FASL)/FAS signaling, thereby decreasing bone resorption and maintaining bone mass (140). Given the potential of IFN-γ treatment to alleviate OVX-induced osteoporosis, Dimitrios Agas et al. utilized IFN-γ gene delivery in OVX mice. This approach circumvents concerns associated with supraphysiologically high doses and allows for sustained efficacy at lower doses over extended durations, unlike conventional IFN-γ recombinant protein injection (195, 210). The results of the study showed that IFN-γ gene delivery caused a dramatic worsening of osteoporosis due to increased release of inflammatory/osteoclastogenic cytokines from bone marrow cells (195). IFN-γ enhanced the IL-17/IL-1/TNF signaling network and accelerated osteoclast-mediated bone resorption (195, 211).

Additionally, it was shown that systemic administration of 1 × 10^6^ IU/kg of IFN-γ three times a week for three weeks exacerbated OVX-induced osteoporosis by promoting bone resorption and bone loss, whereas silencing of IFN-γ significantly ameliorated OVX-induced osteoporosis (138, 139). Michaela Robbie Ryan et al. found that OVX-induced estrogen loss enlarged the size of the T-cell pool (212). OVX mice exhibited diminished TGF-β expression, leading to heightened IL-7 and IFN-γ expression. Expression of IL-7 and IFN-γ is reciprocal, so silencing either factor significantly ameliorates OVX-induced osteoporosis (212). Therefore, under estrogen-deficient conditions, IFN-γ promotes OC formation and bone loss more than it directly resists OC formation (212). The net balance between the direct antiresorptive activity of IFN-γ and its proresorptive effects on OC formation and bone resorption was deviated (138, 139, 212). For the mechanism and cause of the bidirectional action of IFN-γ on OC refer to 4.4 Effects of IFN-γ on bone mass. In conclusion, in postmenopausal estrogen-deficient osteoporosis, IFN-γ can cause increased bone loss by regulating OC formation, but the specific pathogenesis and signaling pathways are not yet fully understood and are not yet available for clinical application.

### 5.2 Crosstalk between IFN-γ and secondary osteoporosis

Glucocorticoids (GCs) have a significant impact on both bone remodeling and immune cells (213). The high concentrations of GCs often present in steroid therapy can lead to catabolic effects on bone, which in turn can increase the risk of osteoporosis. In fact, GC-induced osteoporosis (GIOP) is widely recognized as the most common cause of secondary osteoporosis (100). GCs have been shown to exert distinct effects on OBs, OCs, and OYs, with the most significant effects being the inhibition of OB differentiation and function, the promotion of OB apoptosis, and the reduction of bone formation (213, 214). Numerous studies have shown that cytokines such as TNF-α, IL-6, and IFN-γ may play a role in regulating glucocorticoid-induced osteoporosis, the exact mechanisms of these interactions have yet to be fully elucidated and warrant further investigation.

Rheumatoid arthritis (RA) is known to cause secondary osteoporosis and fragility fractures (215). We explored the effect of IFN-γ on secondary osteoporosis by cutting through the effect of IFN-γ on RA. Bone loss is closely correlated with ACPA in RA that has already developed (216). During OB differentiation, Bettina Groetsch et al. demonstrated a dual role for ACPA and IFN-γ signaling, with the observed changes being connected to various cellular processes during OB differentiation, fusion, and maturation. Co-stimulation of OCs by ACPA and IFN in the inflamed synovium of RA patients may function as a negative feedback loop to regulate the fusion of mature OCs (170). A unique idea for treating RA-mediated secondary osteoporosis involves targeting these two pathways based on the disease stage in RA patients.

More and more potential therapeutic agents to control secondary osteoporosis by inhibiting the expression of IFN-γ have entered the picture over the years. A study by Xilan Yang and colleagues revealed that treatment with glyburide significantly reduced the expression levels of IFN-γ, TNF-α, and IL-6, resulting in increased bone callus volume and bone volume fraction. Additionally, there was a decrease in the number of OC in the bone-chondral interface, and an improvement in the maximum torque and yield torque of fractures (217). It is thus speculated that glyburide may be used as a potential candidate for treating diabetes-induced osteoporosis. Yasir Akhtar Khan et al. found that C-FhHDM-1 inhibited CIA-induced expression of TNF, IL-17 and IFN-γ in the joints and showed that C-FhHDM-1 could be an adjuvant therapy for the prevention of osteoporosis caused by RA (218). Erik Biros et al. showed that D-tryptophan successfully inhibited monocyte polynucleation and reduced OC production in osteoporosis in the presence of IFN-γ *in vitro*. This amino acid was established as a viable therapeutic candidate to be studied in patients with osteoporosis (19).

## 6 Summary

Osteoporosis, a common metabolic bone disease, occurs when osteoblast-mediated bone formation is lower than osteoclast-mediated bone resorption, resulting in disturbed bone remodeling. To date, osteoporosis prevention and treatment remains a challenge. Primary osteoporosis has been treated with hormone replacement therapy and selective androgen receptor modulators (SARMs), but long-term use has been associated with increased rates of heart disease, stroke, and breast cancer and prostate cancer (219). Currently, bisphosphonates are recommended as the clinical first-line treatment for osteoporosis (primary and secondary) (220). However, numerous studies show that treatment with bisphosphonates is associated with an increased incidence of osteonecrosis of the jaw, atypical osteonecrosis of the femur, and malignancy (221, 222). Therefore, new, effective drugs or therapies are necessary for the long-term relief and treatment of osteoporosis.

In the past 20 years, a new interdisciplinary field called osteoimmunology has emerged, which aims to investigate the relationship between the immune system and bone. The effect of various cytokines on bone in immunosteology promotes clinical research on new therapies for relevant diseases. IFN-γ acts in bone homeostasis by activating complex signaling pathways, enhancing the formation of OBs *in vivo* but inhibiting the formation of bone marrow adipocytes. IFN-γ exerts a dual effect on osteoclastogenesis, wherein it impedes the early differentiation of OCs by selectively modulating the RANK-RANKL signaling pathway, while concurrently facilitating the fusion of mononuclear OC precursors during the advanced stages of OC formation. Nevertheless, in medical conditions such as postmenopausal osteoporosis, infection, and inflammation, the suppressive effect of IFN-γ on OCs was observed to be more pronounced than its indirect stimulatory effect. IFN-γ exerts different effects at different stages of osteoporosis.

Currently, IFN-γ is not used clinically for the treatment of osteoporosis. The therapeutic potential of IFN-γ in osteoporosis is modulated by various factors, including but not limited to the specific dosage, frequency, administration route, and duration of treatment, highlighting the importance of carefully considering these variables in designing IFN-γ-based therapeutic strategies. To develop effective therapeutic strategies for osteoporosis using IFN-γ, it is critical to investigate the impact of IFN-γ on both the skeletal and immune systems, while taking into account the underlying pathological mechanisms of the disease, and carefully evaluating the optimal dosage, frequency, administration route, and duration of treatment. Doing so can minimize the risk of counterproductive effects and potentially improve the efficacy of IFN-γ-based therapies for osteoporosis.

In future studies, the focus should be on the molecular pathways and conditions associated with the different functions of IFN-γ in osteoporosis from the perspective of osteoimmunology. For example, IFN-γ plays a dual role in OC differentiation. Most studies suggest that IFN-γ acts at multiple sites of the RANKL/RANK/OPG signaling pathway (RANK, TRAF6 and NFATc1) (130). Identifying the key initial sites is crucial for this stage of the study. Second, considering the great differences in the pathogenesis of primary and secondary osteoporosis, the therapeutic effects of IFN-γ need to be investigated by evaluating the dose, frequency, usage, and duration of IFN-γ treatment separately. It is important to investigate the role of IFN-γ according to the pathogenesis of osteoporosis (19). In the case of primary osteoporosis, IFN-γ seems to play different roles at various stages of pathogenesis. Therefore, finding the right time to use and exploring the role of IFN-γ in the bones and immune system of patients with primary osteoporosis may be important.

## Author contributions

SL: Investigation, Methodology, Writing – review & editing, Project administration, Validation, Writing – original draft. GL: Supervision, Writing – review & editing, Investigation, Methodology. SH: Writing – review & editing, Funding acquisition, Supervision.

## Glossary

**Table:**

APC	Activated protein C
NFATc1	Nuclear factor of activated T cells cytoplasmic 1
ALP	Alkaline phosphatase
AD	Alzheimer’s disease
ACPA	Anti-citrullinated protein antibody
RGD	Arginine-glycine-aspartic acid
Axin	Axis inhibition protein
BMUs	Basic multicellular units
BMP	Bone morphogenetic protein
BRC	Bone Remodeling Compartment
BSP	Bone sialoprotein
CK1α	Casein kinase 1α
CTSK	Cathepsin K
CTSL	Cathepsin L
C/EBPα	CCAAT/enhancer binding protein alpha
JNK	C-Jun N-terminal kinase
CIA	Collagen-induced arthritis
CTL	Cytotoxic T lymphocyte
DMP1	Dentin matrix protein 1
DC	Dentritic cell
DOP	Diabetic osteoporosis
DKK	Dickkopf
ERK	Extracellular-signal-regulated kinase
c-Fos	Fos proto-oncogene
FZD	Frizzled
SFRP1	Frizzled-related protein 1
GIOP	Glucocorticoid-induced osteoporosis
GCs	Glucocorticoids
GSK3β	Glycogen synthase kinase 3beta
GRB2	Growth factor receptor-bound protein 2
HSPCs	Hematopoietic stem and progenitor cells
HSCs	Hematopoietic stem cells
IFN-γ	Interferon-γ
IFNγR	IFN-γ receptor
IHD	Inflammatory heart disease
IGF	Insulin-like growth factor
IL	Interleukin
IOF	International Osteoporosis Foundation
JAK	Janus kinase
LRP4/5/6	Lipoprotein receptor-related protein 4/5/6
LEF	Lymphoid enhancing factor
M-CSF	Macrophage-colony stimulating factor
M-CSF-R	Macrophage-colony stimulating factor receptor
MHC	Major histocompatibility complex
MMP-13	Matrix metalloproteinase-13
MSCs	Mesenchymal stem cells
MITF	Microphthalmia-associated transcription factor
MAPK	Mitogen-activated protein kinase
MCP	Monocyte Chemoattractant Protein
NK	Natural Killer
NF-κB	Nuclear factor kappaB
OA	Osteoarthritis
OB	Osteoblast
OCN	Osteocalcin
OC	Osteoclast
OY	Osteocyte
OP	Osteoporosis
OPG	Osteoprotegerin
OVX	Ovariectomized
PTH	Parathyroid hormone
PBMCs	Peripheral blood mononuclear cells
PPARγ	Peroxisome proliferator-activated receptor gamma
PI3K	Phosphoinositide 3-kinase
Dsh	Phosphoprotein Dishevelled
p-GSK3β	Phosphorylated glycogen synthase kinase-3 beta
P-β-catenin	Phosphorylated β-catenin
PGE2	Prostaglandin E2
PKA	Protein kinase A
Akt	Protein kinase B
PKC	Protein kinase C
PKR	Protein kinase R
PP1A	Protein phosphatase-1-catalytic subunit alpha
PU.1	Purine Rich Box-1
RANK	Receptor activator of nuclear factor-kappa B
RANKL	Receptor activator of nuclear factor-kappa B ligand
ROS	Reactive oxygen species
RA	Rheumatoid arthritis
Runx2	Runt-related transcription factor 2
SOST	Sclerostin
sFRP-1	Secreted frizzled-related protein-1
STAT1	Signal transducer and activator of transcription 1
TRAP	Tartarate-resistant alkaline phosphatase
TCF	T-cell factor
TRAFs	TNF receptor-associated factors
TAZ	Transcriptional Co-Activator With PDZ-Binding Motif
TGF	Transforming growth factor
TNF	Tumor necrosis factor
COL1A	Type I collagen alpha
WR	Wheel-running
Wnt	Wingless
